# Extraosseous aneurysmal cyst in hand: a case report

**DOI:** 10.1186/1757-1626-1-268

**Published:** 2008-10-24

**Authors:** Ajay Sahu, Sarabjeet S Gujral, Sanjeev Gaur

**Affiliations:** 1 Gandhi Medical College, Bhopal, India; 2 Current address : Patel ward, Gandhi Medical College Hospital, Bhopal, India; 3 Current address : Ward D1, Stepping Hill Hospital, Stockport, Manchester, UK

## Abstract

**Background:**

The presence of primary aneurysmal cyst in soft tissues is a extremely rare and its presence in the soft tissues of hand has never been reported in literature before. We report the first ever case of extraosseous aneurysmal cyst in hand.

**Case Presentation:**

A twelve years old girl presented with a swelling in the thenar region on palmer aspect of right hand growing slowly since three months. On X ray, CT scan and excision biopsy the lesion was found to be separate from bone and located in the soft tissue. Its diagnosis was confirmed on histopatholgical examination.

**Conclusion:**

Previously few authors have reported extraosseous aneurysmal cyst in the soft tissues of shoulder, hip and pelvic girdle but nobody has reported its presence in the soft tissues of hand.

## Introduction

Aneurysmal bone cyst is defined as a lesion of bone characterised by the presence of spongy or multi locular cystic tissue filled with blood. The presence of aneurysmal cyst in soft tissues is a rare phenomenon and its presence in the soft tissues of hand has never been reported before.

## Case presentation

A twelve years old girl presented with slow growing swelling in the thenar palmer aspect of the right hand since three months. It was not accompanied by pain. There was no history of trauma. On palpation a bony hard, immobile mass, measuring 4.5 cm. in diameter was felt on the thenar eminence. It was non-tender and was not fixed to underlying bone.

**Figure 1:**
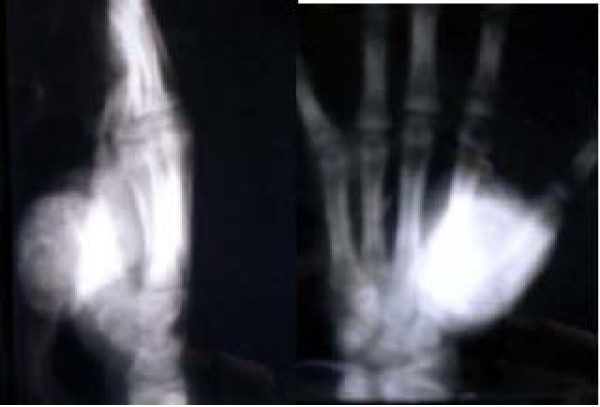
AP & Lateral radiograph of hand of our patient

**Figure 2:**
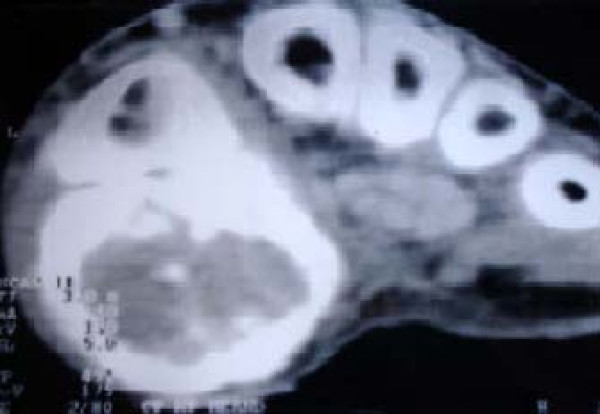
Axial view of hand by CT scan

**Figure 3:**
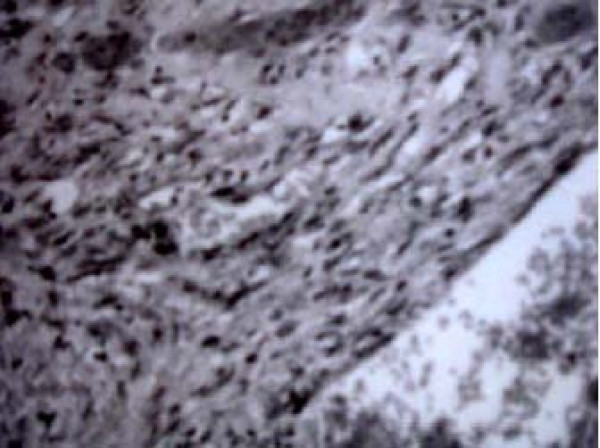
Histopathological aapearance of the lesion under microscope

The X ray showed a lytic lesion with sharply circumscribed bony outline and accompanying mild periosteal reaction in the first metacarpal region (fig. 1). A subsequent CT scan also showed a lytic lesion with bony outline separate from first metacarpal (fig. 2). The lesion was reported as a calcifying hematoma. Excision biopsy was performed and it was noticed intraopertively that the growth was in the soft tissue and there was no connection with the bone. On histopathological examination it was diagnosed as an extraosseous aneurysmal cyst (fig. 3). The patient was followed up for twenty-four months and there was no recurrence of the lesion.

## Discussion

Aneurysmal bone cyst arises usually as a primary benign tumour of bone. Lesion is usually primary and less commonly secondary, superimposed on pre-existing benign or malignant bone lesions [1].

There are very few instances of aneurysmal cyst reported in soft tissue. Aneurysmal bone cyst is an infrequent finding in hand [2]. Two authors have reported extraosseous aneurysmal cyst in the shoulder region with no connection to bone [3,4]. Other aneurysmal cysts were reported in the soft tissue surrounding the hip [5] and in the left retroclavicular soft tissue [6]. More recently aneurysmal cyst has been reported in the pelvic girdle region [7] whereas most previous cases were related to the pectoral region. They found the aneurysmal cyst in the hip adjacent to left illiac bone. One author has commented on the pathogenesis of extraosseous aneurysmal cyst that it is not clear but it can be considered as a result of reparative process stemming from an unperceived trauma.[3]

## Conclusion

A thorough literature search and an exhaustive online search revealed no reported cases of aneurysmal cyst in the soft tissue of hand. If we encounter it in unusual sites like hand then it should be diagnosed based on its histology. Depending on symptoms we would advocate it to be treated with an excision surgery.

## List of abbreviations used

CT scan: Computerised Tomographic scan.

## Consent

"Written informed consent was obtained from the patient for publication of this case report and accompanying images. A copy of the written consent is available for review by the Editor-in-Chief of this journal."

This is to confirm that the patient has given her informed consent for the case report to be published.

## Competing interests

The authors declare that they have no competing interests.

## Authors' contributions

AS analyzed and interpreted the patient data regarding the presentation and writing the report. SSG performed the literature search and wrote the discussion. SG performed the excision biopsy. He also helped in writing the clinical presentation and conclusion. All authors read and approved the final manuscript.

## Disclosure

The authors did not receive any outside funding or grants in support of their research for or preparation of this work. Neither they nor a member of their immediate families received payments or other benefits or a commitment or agreement to provide such benefits from a commercial entity. No commercial entity paid or directed, or agreed to pay or direct, any benefits to any research fund, foundation, division, center, clinical practice, or other charitable or nonprofit organization with which the authors, or a member of their immediate families, are affiliated or associated.

Stepping Hill Hospital, Stockport NHS Foundation Trust, Stockport, UK
